# A community-based study of hypertension and cardio-metabolic syndrome in semi-urban and rural communities in Nigeria

**DOI:** 10.1186/1472-6963-10-71

**Published:** 2010-03-19

**Authors:** Ifeoma I Ulasi, Chinwuba K Ijoma, Obinna D Onodugo

**Affiliations:** 1 Renal Unit, Department of Medicine, College of Medicine, University of Nigeria Teaching Hospital, Enugu, Nigeria

## Abstract

**Background:**

The prevalence of cardio-metabolic syndrome (CMS) is increasing worldwide. In people of African descent, there is higher prevalence of hypertension and complications than other races. Bearing in mind these facts, we looked at the CMS in the general population and the population with hypertension. Using the new International Diabetes Federation (IDF) definitions of CMS, we studied its prevalence in semi-urban and rural communities in South-east Nigeria in relation to hypertension.

**Method:**

This is a cross sectional population based study involving 1458 adults aged from 25 to 64 years. Diagnosis of CMS was based on the new IDF criteria using the anthropometric measurements for Europids as there is none yet for blacks. Hypertension was defined according to the WHO/ISH criteria.

**Results:**

The overall prevalence of CMS was 18.0% in the semi-urban community as against 10.0% in the rural community increasing to 34.7% and 24.7% respectively in the population with hypertension. The prevalence of co-morbidities - hyperglycaemia, abdominal obesity, and hypertriglceridaemia were 13.9%, 41.1% and 23.9% while in the hypertensive populations they were 21.2%, 55.0% and 31.3% in the general population in both communities combined. Except for low HDL cholesterol, every other co-morbidity was higher in hypertensive population than the general population.

**Conclusion:**

The high prevalence of CMS in the semi-urban population especially for the population with hypertension underscores the double burden of disease in developing countries. The lesson is while infections and infestations are being tackled in these countries the non-communicable diseases should not be neglected.

## Background

The prevalence of cardio-metabolic syndrome (CMS) is increasing worldwide; indeed it's occurrence has been described as a global epidemic [1]. The Third Report of the National Cholesterol Education Program Expert Panel (NCEP) on Detection, Evaluation, and Treatment of High Blood Cholesterol in Adults (ATP III) found that ethnicity influences the prevalence of metabolic syndrome [2]. Mexican Americans are now known to have the highest age-adjusted prevalence of metabolic syndrome for both men and women, and African-American women have a higher incidence of metabolic syndrome than African-American men [2,3]. Also, in people of African descent there is higher prevalence of hypertension and complications than other races [4].

Current knowledge suggests the importance of increased body mass index (BMI) especially visceral fat in the pathophysiology of hypertension [5]. Some recent studies suggested that CMS can predict the development of hypertension [6]. CMS is characterized by the cluster of physical and biochemical abnormalities in an individual which predisposes the person to increased risk of developing cardiovascular disease and it is also predictive of future diabetes mellitus [7]. CMS and some of its components are related to diet [8]. The average staple diet of the rural African population consists mostly of carbohydrate (70%) with rather low protein intake. It has high fibre content, moderate oil mostly in form of palm oil and average salt. In the semi-urban population many have adopted western diet including fast foods which is high in carbohydrate and salt, moderate protein and has average fibre content.

It is important therefore to investigate the presence of CMS in patients with hypertension who may indeed be at risk of diabetes mellitus in the future as well as its association with increased cardiovascular morbidity and mortality. Bearing in mind these facts; we looked at the CMS in the general population and the population with hypertension. Using the new International Diabetes Federation (IDF) definitions of CMS, we studied its prevalence in semi-urban and rural communities in South-east Nigeria.

## Method

### Study Area

Nigeria is made up of 6 geo-political zones with a total of 36 states and the Federal Capital Territory. The South East zone comprises of 5 states (Enugu, Ebonyi, Imo, Abia and Anambra) with estimated population of 30 million people. They are Igbo speaking and the zone is located between latitude 5° - 7° North of the Equator and longitude 6° - 8° East Greenwich.

The study area is Ujodo Nike Local Government Development Centre (LGDC) in Enugu North Local Government Area (LGA) of Enugu State. This LGDC comprises of three communities namely Mbulu-Ujodo, Mbulu-Awuli and Emene communites. Emene-Nike was chosen as the only semi-urban area and Mbulu-Ujodo was randomly selected from the two rural communities. Mbulu-Ujodo comprises of villages or hamlets viz.: Obinagu, Nchantancha, Akpuoga and Onuogba.

### Study Population

In the rural community many young people have migrated to the cities leaving a preponderance of relatively elderly population. They are mostly of low socio-economic status engaging mainly in farming. In the semi-urban community most inhabitants in the study area are individuals with low socio-economic status mainly artisans, traders and low income workers who live in over-crowded homes with poor sanitary conditions. This study population by culture and ancestry believe in the healing powers of their gods and their chief priests and the native doctors hence the use of native medications is very common. Most individuals would try alternative medicine and spiritual healing homes before presenting to hospitals [9]. Apart from consulting native doctors/healers, patronage of patent medicine dealers and laboratory scientists is rampant. This is because of inability to afford cost of health care services. In the Igbo tribe, being overweight/obese is preferred and use of mercury containing soaps and bleaching cream to achieve fair skin complexion is encouraged as these features, are seen as marks of beauty [10].

### Sample size, Frame, Design and Selection

A minimum target sample size of 2000 adults aged 25 to 64 stratified by sex and 10-year age group as per modified WHO STEPS[11] surveillance and Program for Detection and Management of Chronic Kidney Disease, Hypertension, Diabetes and Cardiovascular Disease in Developing Countries (KHDC)[12] questionnaires was used. To compensate for anticipated non-response or attrition, the number was projected to 2200.

Emene, Nike has a population of 70,000 and Mbulo-Ujodo, Nike has a population of 40,000. Since the current census list (2006 census list) was not yet in public purview, enumeration was done. Two thirds were selected from the semi-urban area and the remaining third from the rural community to achieve proportional representation.

### Study Personnel

The personnel were made up of doctors, nurses, medical students, social workers, sociologists and laboratory technologists drawn from the staff of Departments of Medicine and Pharmacology, College of Medicine, University of Nigeria Teaching Hospital (UNTH), Enugu, Nigeria.

### Questionnaire and Consent form

The questionnaire was a hybrid of the WHO STEPS [11] and the KHDC[12] questionnaires with some additional questions to address the peculiarities in the locale of the study. The questionnaire was translated to Igbo language, the prevailing language spoken in the study area. Self-reported history of alcohol consumption, use of tobacco as snuff or cigarette or any other form of tobacco as well as family, present and past histories of hypertension, diabetes mellitus, renal disease and stroke/heart attack were obtained from the respondents.

### Ethical Review

The project proposal, the modified questionnaire and the consent form were reviewed and approved by the University of Nigeria Teaching Hospital, Enugu, Ethics Review Committee.

### Clinical Evaluation and Collection of Samples

The Clinical evaluation and collection of samples were carried out at screening centres set-up at spots convenient for the participants: churches, market square, town hall and the Health Centre at Akpuoga, Nike.

The participants' anthropometric data were taken: height, weight, waist and hip circumferences. The weight was measured using standardized bathroom scale (10 kg weight was used). The heights were measured with a standiometer. The waist circumference was taken at mid-way between the sub-costal margin and the iliac crest while the hip circumference was taken at the widest diameter. The blood pressure measurements were taken three times in the arm with the participants sitting using Accusson's mercury sphygmomanometer with appropriate cuff sizes. Three milliliters of fasting blood was taken into plain bottle for blood chemistry (lipids) -total cholesterol (TC), low density lipoprotein cholesterol (LDL-C), high density lipoprotein cholesterol (HDL-C) and triglycerides (TGs). Blood sugar was done with Accuchek glucometer. All samples were analyzed at the Dept of Medicine Research Laboratory, University of Nigeria Teaching Hospital, Enugu. The TC and TGs were measured by enzymatic colorimetric method and HDL- C and LDL-C by differential precipitation enzymatic colorimetric using semi-autoanalyser Mitra Photometer (Linear Chemicals S.L., Barcelona, Spain). All parameters were expressed as mmol/l.

### Definitions

Diagnosis of CMS was based on the new International Diabetes Federation (IDF) criteria[13] using the anthropometric measurements for Europids as there are none yet for blacks. As defined by IDF: central obesity - waist circumference ≥ 94 cm for men and ≥ 80 cm for women. Plus any two of the following factors:

• raised TG level - ≥ 1.7 mmol/l or specific treatment for this lipid abnormality

• reduced HDL - cholesterol - < 1.03 mmol/l in males and < 1.30 mmol/l in females or specific treatment for this lipid abnormality

• raised blood pressure - systolic ≥ 130 mmHg or diastolic ≥ 85 mmHg or treatment of previously diagnosed hypertension

• raised fasting blood sugar - ≥ 5.6 mmol/l or previously diagnosed type 2 DM

Hypertension was defined as systolic blood pressure ≥ 140 mmHg or diastolic blood pressure ≥ 90 mmHg or both and/or concomitant use of antihypertensive medications according to the WHO/ISH guidelines[14].

### Statistical Analysis

The Statistical Package for Social Sciences (SSPS Inc, Chicago, IL) version 16 statistical software was used for data analysis. Cross tabulation was used to determine the prevalence of metabolic syndrome and the individual components of metabolic syndrome in the communities. We examined the relationship between the various variables such as sex, age, level of education or years in school, site (semi-urban or rural community), abdominal obesity, present history of hypertension and DM in both communities. The use of tobacco was examined by using 'ever used' or 'never used' tobacco status. And we assessed alcohol consumption by number of drinks taken (<2 or >2).

All tests were two-tailed with p < 0.05 taken as statistically significant.

## Results

A total of 1939 were analyzed out of 2182 individuals who were screened. The others could not be analyzed because of incomplete data. Of the 1939 participants, about 500 female participants were removed to avoid skewing the data leaving a total of 1458. This was done by removing the women recruited towards the end of the study.

### Demographic Characteristics

Table 1 shows the demography of the study population including the mean ages of the participants in the rural and semi-urban communities. The mean age for study population was 43.8 ± 13.7 years (male respondents - 44.1 ± 14.2 years; female respondents - 43.4 ± 13.2 years, p = 0.35). The mean age of the participants with CMS (48.1 ± 11.1 years) was significantly higher than that of the participants without CMS (42.8 ± 13.9 years), p < 0.001.

**Table 1:** Demographic characteristics of the study population

Characteristics	Semi-urban (1088)	Rural (370)	All (1458)	p-value
Men (%)	49.6	45.7	48.6	0.188
Mean age	42.3 ± 13.5	48.2 ± 13.2	43.8 ± 13.7	<0.001*
Age group(%):	-	-	-	-
25 - 34 years	36.5	20.0	32.3	-
35 - 44 years	17.5	14.1	16.6	<0.001*
45 - 54 years	21.6	27.9	22.4	-
55 - 64 years	24.4	41.4	28.7	-
Number of years in School	-	-	-	-
(%):	-	-	-	-
No formal schooling	14.5	55.1	24.8	-
≤ 6 years	21.7	25.1	22.6	-
7 - 12 years	32.9	15.9	28.6	<0.001*
>12 years	30.9	3.8	24.0	-

*- p-value: significant

### Prevalence of Hypertension and CMS

Table 2 shows the characteristics of the study population - semi-urban and rural communities. The overall prevalence of CMS was 18.0% in the semi-urban community as against 10% in the rural community increasing to 34.7% and 24.7% respectively in the population with hypertension. The prevalence of hypertension was 35.4% in the semi-urban community as against 25.1% in the rural community. Among all participants, prevalence of CMS and hypertension were 15.9% and 32.8% respectively.

**Table 2:** Anthropometric characteristics, Physical Activity and Prevalence of CMS and Hypertension in study population

	Semi-urban (1088)	Rural (370)	All (1458)	p-value
BMI (%):	-	-	-	-
≥ 25 to <30 kg/m^2^	33.1	27.1	31.6	0.032*
≥ 30 kg/m^2^	19.2	11.7	17.3	0.001*
Waist circumference (%):	-	-	-	-
≥ 80 cm female	70.7	53.7	66.0	<0.001*
≥ 94 for male	16.7	8.9	14.9	0.014*
Physical activity (%)	94.5	99.1	95.8	<0.001*
MS	18.00	10.00	15.9	<0.001*
Hypertension	35.4	25.1	32.8	<0.001*

*- p-value: significant

### Prevalence of Components of CMS

The prevalence rates of CMS and other co-morbidities such as abdominal obesity, hyperglycaemia (fasting blood sugar ≥ 5.6 mmol/l), hypertriglyceridaemia and low HDL cholesterol in the general and hypertensive populations in the communities are as shown in Table 3. Except for low HDL every other co-morbidity was higher in hypertensive population than the general population. Overall, blood pressure (using cut-off for CMS) had the highest prevalence being 42.3%, followed by abdominal obesity - 41.1%. The hypertensive population as expected had significantly higher prevalence rates than the general population except for low HDL-C.

**Table 3:** Prevalence of co-morbidities in non-hypertensive, hypertensive and general populations in the various communities

		MS	Hyperglycaemia (≥ 5.6 mmol/l)	Abdominal obesity	^$^**TC> >6.2 mmo**l	*TG ≥ >1.70 mmol	^#^**HDL**
**Rural**	NHP	5.1	12.2	20.1	4.7	13.7	33.9
	HP	24.7	23.0	34.4	9.7	22.6	41.3
	GP	10	14.9	28.2	5.9	15.9	35.8
	ORs	6.12	2.15	1.49	2.18	1.83	1.37
	(95% CI)	(3.02 - 12.62)	(1.15 - 3.99)	(0.90 - 2.46)	(0.90 - 5.27)	1.01-3.32	(0.84 - 2.22)
	p-value	<0.001	0.014	0.12	0.79	0.043	0.20
**Semi- urban**							
	NHP	8.8	9.6	35.2	5.7	23.1	33.8
	HP	34.7	20.7	57.1	10.8	33.4	27.7
	GP	18.0	13.6	43.7	7.5	26.7	31.7
	Ors (95% CI)	5.52 (3.94 - 7.74)	2.45 (1.69 - 3.57)	2.66 (2.07 - 3.45)	1.99 (1.26 - 3.16)	1.67 (1.26 - 2.21)	0.75 (0.57 - 0.99)
	p-value	<0.001	<0.001	<0.001	0.003	<0.001	<0.043
**Both communities**	NHP	7.7	10.4	34.3	5.4	20.4	33.9
	HP	32.8	21.2	55.0	10.6	31.3	30.4
	GP	15.9	13.9	41.1	7.1	23.9	32.7
	Ors (95% CI)	5.82 (4.30 - 7.89)	2.32 (1.69 - 3.18)	2.35 (1.88 - 2.94)	2.06 (1.37 - 3.09)	1.78 (1.38 - 2.28)	0.85 (0.67 -1.09)
	p-value	<0.001	<0.001	<0.001	<0.001	<0.001	0.20

Key:^$^- total cholesterol; *- triglyceride; ^# ^- 1.03 mmol/l in males & <1.3 mmol/l in females; NHP non-hypertensive population; HP- population with hypertension; GP- general population; *- p-value: significant

**Figure 1:**
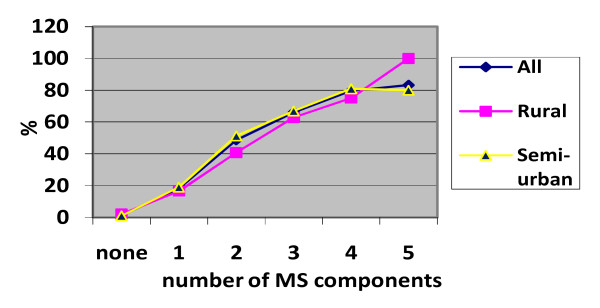
**Prevalence of hypertension by number of components of MS**.

The prevalence of main population health determinants (which are also part of the natural history of CMS and hypertension) such as tobacco use, alcohol consumption and physical inactivity are as displayed in Table 4. The prevalence of CMS increased up until age 44 - 54 years and then declined while the prevalence of hypertension showed continuous increase to age group 55 - 64 years. The prevalence of hypertension increased with the number of MS risk factors, Fig. 1.

**Table 4:** Prevalence of population health determinants in general and hypertensive populations in the communities

Health determinants	Semi	Urban	Community %	ORs (95% CI)	P-value	Rural	Community	%	ORs (95% CI)	p-value
	NHP	HP	GP			NHP	HP	GP		
Tobacco use	12.3	18.9	14.6	1.65 (1.15 - 2.39)	0.007*	14.8	17.7	15.5	1.24 (0.64 - 2.44)	0.535
Alcohol use	61.9	55.8	59.9	0.78 (0.58 - 1.03)	0.083	64.9	69.8	66.1	1.25 (0.68 - 2.13)	0.473
Inactivity	4.0	8.4	5.5	0.45 (0.26 - 0.79)	0.004*	0.7	1.1	0.82	0.67 (0.06 - 7.47)	0.741

Key: NHP - non-hypertensive population; HP - population with hypertension; GP - general population; * - p-value: significant

## Discussion

### Demographic and Anthropometric Characteristics

The study has shown a larger proportion of young people (age group 25 - 34 years) - 36.5% in the semi-urban area while the older people were more predominant in rural community (age group 55 - 64 years) - 41.4%. This age disparity in population distribution reflects the trend of rural-urban migration that is common in Nigeria. The semi-urban community recorded a higher percentage for the two indices for assessment of obesity i.e. waist circumference and BMI (female 70.7%, male 16.7% and 19.2% respectively) than for the rural community (female 53.7%, male 8.9% and 11.7%). This is a reflection of the adoption of western life style in semi-urban community compared to the rural community. A study in Seychelles by Kelliny et al noted: female 75.6%, male 35.8% for waist circumference and 26.9% for BMI [15]. While in Cameroun, Fezeu at al documented prevalence rates of 20.5% and 10.9% of obesity using BMI in urban and rural populations respectively [16].

### CMS in Rural versus Semi-urban Communities

Our study has demonstrated a high prevalence of cardio-metabolic syndrome in the semi-urban community (18.0%) though not as high as in many developed countries. This observation is of concern. The prevalence of CMS in the semi-urban community is about double that recorded for the rural community (10.0%). Information on population-based study on CMS in Africa is sparse. However, Fezeu et al [16] recorded much lower values than in our study: 1.8% for rural community and 5.9% for urban community. The higher value recorded in our study may suggest that Nigeria is in transition phase between the traditional African culture and western culture. A study in South Africa [17] looking at corporate executives found a prevalence of 31% using ATP III. Another study in the US, of African Americans aged 35-84 years, recorded a prevalence of metabolic syndrome of 43.3% in women and 32.7% in men [18]. Studies in US [19], and Europe [20] have documented 34%, and 29.6% respectively.

The finding of high percentage of CMS in this study is of clinical and public health importance as metabolic syndrome is an important risk factor for diabetes, coronary heart disease, stroke and CKD. Furthermore, in Nigeria like in most developing countries public awareness of the dangers western life style pose to health is limited. Studies in some other developing countries have noted high prevalence of CMS similar to or even higher than what obtains in developed countries [7,15,21]. In Chile, they noted a prevalence of 31.6% for general population and 61.6% for hypertensive population [7]. These are way above 15.9% and 32.8% noted respectively for general and hypertensive populations in this present study. Albeit, the implication of this trend in developing countries is ominous because of the poor state of health services and associated high prevalence of communicable diseases. These bring the double burden of disease as emphasized by World Health Organization to the fore [22].

### Prevalence of Components of CMS

Visceral fat has been described as an endocrine organ secreting adipocytokines which are aetiologically related to CMS [23] and hypertension [24]. Apart from blood pressure which had a prevalence of 42.3%, abdominal obesity had a high prevalence of 41.1%. In Cameroun, Fezeu et al [16] identified central obesity as the key determinant factor for CMS in that population. However, in Chile [7], they noted higher prevalence of 46% for elevated blood pressure and 29% for abdominal obesity. We used IDF definition of CMS in this present study while the Chilean study used the updated ATP III. This could in part explain the difference but in Seychelles, they documented 55.8% and 61.5% using IDF and ATP III definitions for abdominal obesity [15].

As expected the prevalence of hypertension increased with age. It is noted in JNC VI that by age 65 years more than two thirds of individuals already have hypertension [25]. On the other hand the prevalence of CMS in this present study increased up until age group 45 to 54 years and then declined. This may be related to the loss of body fat with aging in this population. Kelliny et al made similar observation but it was only in the male population using ATP III definition [15]. Most other studies show continuous increase of prevalence with age [19-21].

### Limitations of the Study

Though this study is population-based, looking at rural and semi-urban communities, there were some limitations. The cross sectional design of the study.

The use of self report for alcohol consumption and use of tobacco, being mostly illiterate and semi-literate population the accuracy of their reporting may be questioned.

## Conclusion

The prevalence of MS in this study was high especially when compared with figures from Cameroun [16], a neighbouring country. This high prevalence of MS in the semi-urban population especially for the population with hypertension underscores the double burden of disease in developing countries. While infections and infestations are being tackled in these countries the non-communicable diseases should not be neglected.

## Competing interests

The authors declare that they have no competing interests.

## Authors' contributions

II, CK, OD made contributions in conception and design of the study. They also contributed in acquisition and interpretation of data. II/CK drafted the manuscript and II/OD revised it. All have given their approval for the version to be published.

## Pre-publication history

The pre-publication history for this paper can be accessed here:

http://www.biomedcentral.com/1472-6963/10/71/prepub
